# Genetic Diagnosis in Movement Disorders. Use of Whole-Exome Sequencing in Clinical Practice

**DOI:** 10.5334/tohm.678

**Published:** 2022-04-20

**Authors:** Patricio Millar Vernetti, María Agustina Ruiz Yanzi, Malco Rossi, Marcelo Merello

**Affiliations:** 1 Movement Disorders Clinic, Neurology Department, Fleni, Buenos Aires, Argentina; 2 Neurology Department, Fleni, Buenos Aires, Argentina; 3 National Council for Scientific and Technical Research (CONICET), Argentina

**Keywords:** WES, whole-exome sequencing, genetics, diagnosis, controversies

Use of next-generation sequencing, including whole-exome sequencing (WES) has not only allowed diagnosis to be reached in patients with atypical phenotypes, but also led to detection of new pathogenic variants, as well as to linking of specific clinical manifestations to known diseases. Nevertheless, controversy persists regarding routine implementation of WES in clinical practice [1,2]. Interpretation of results and understanding of the clinical relevance can be problematic, particularly in cases of variants of unknown significance (VUS) [3]. Application of WES can however be cost-effective when based on appropriate clinical criteria, and may even reduce costs by limiting unnecessary complementary studies [4]. Although slowly becoming more financially accessible, WES remains an expensive study. Inappropriate or indiscriminate use may increase overall healthcare costs, without providing significant benefit. In addition, WES does not detect large gene deletions or duplications, or expansion disorders such as triplet repeat expansions, or genes located on non-coding segments of the genome (introns), and therefore is not useful to diagnose diseases caused by these particular mutations [1,2]. The diagnostic yield of WES in different case series of adult patients with neurological diseases has consistently been around 30% [5,6]. In children, similar [7,8,9], or somewhat higher values have been reported [10]. More specifically in movement disorders, a study including 378 patients with atypical or combined phenotypes, found the diagnostic yield was 22% [11].

In this context, we set out to analyze the indications for WES, through the analysis of results obtained in a cohort of 2948 patients consulting the Movement Disorders clinic at our institution in Buenos Aires, Argentina between July 2015 and July 2019. Prior to indicating the test, all patients had undergone thorough clinical evaluation by a movement disorder specialist, as well as routine diagnostic workup to exclude frequent disorders treated at our tertiary clinic. WES was ultimately indicated based on patient phenotype in 54/2948 (1.8%) patients, lacking definitive diagnosis after exhaustive clinical examination as well as standard blood, urine, and imaging studies, including selective genetic testing for Huntington’s disease, Friedreich’s ataxia, spinocerebellar ataxias due to repeat expansions and DYT1 gene mutations. WES was also requested in cases of combined or atypical phenotypes presenting ataxia, parkinsonism, chorea, tremor, dystonia and spastic paraplegia, and/or patients with positive family history, or early onset of disease.

Of the 54 patients in which WES was obtained,14 (25.9%) presented pathogenic variants and 18 (33.3%) VUS. In 27 (50%), genetic tests for common repeat expansions inducing movement disorders were negative. Main clinical and genetic characteristics are summarized in ***Table 1***. Similar to other results reported in the literature on the diagnostic yield of WES [5,6], in this series of 54 patients, 17 variants were considered clinically pathogenic (35.2%). TRIO analysis was conducted in 7 of 32 patients with genetic findings (21.9%); and proved useful to confirm pathogenicity, in cases where compound heterozygous mutations were present (cases 22, 28, 29). In case 31, in which no family history of autosomal dominant dystonia was detected, TRIO analysis led to a diagnosis in the mother, rather than confirmation of pathogenicity of the mutation. The number of variants interpreted as pathogenic according to phenotype were: 7/19 (36.8%) in ataxia; 4/18 (22.2%) in parkinsonism; 3/3 (100%) in tremor, 1/7 (14.3%) in spastic paraplegia and 6/21 (28.6%) in dystonia. In two patients presenting chorea, no pathogenic variants were identified.

**Table 1:** Findings in patients evaluated with WES: phenotype, age at time of study, genetic test results, homo or heterozygosity, family history, and treatment.


PHENOTYPE/PATIENT	ATAXIA	CHOREA	PARKINSONISM	TREMOR	SPG	DYSTONIA	AGE AT WES (YRS.)	LATENCY FROM THE ONSET OF SYMPTOMS (YRS.)	FINDING	INTERPRETATION	VARIANT	DIAGNOSIS	AVAILABLE TREATMENT	HOMOZYGOUS OR HETEROZYGOUS	FAMILY HISTORY

1			+				27	5	V/V	V/V	FBN1 c.8149G>A(p.Glu2717Lys) + TSC2 c.2245C>T(p.Arg749Trp)	.	NO	Heterozygous	NO

2						+	65	5	V	V	KMT2B c.2068G>C((p.Glu690Gln)	DYT28	GPi DBS [19]	Heterozygous	NO

3					+		66	14	V	V	SETX c.628A>G (p.Ile210Val)	ALS type 4	NO	Heterozygous	NO

4						+	31	13	V	V	ITPR1 c.5149C>A (p.Leu1717Met)	.	NO	Heterozygous	NO

5	+		+				70	10	V	V	GRN c.421G>A (p.Val141Ile)	FTD	NO	Heterozygous	NO

6	+		+				72	2	V	V	CCDC88C c.2491G>A (p.Glu831Lys)	.	NO	Heterozygous	YES (father)

7						+	25	2	V	V	COL4A1 p.(Glu1539Gly)	.	NO	Heterozygous	NO

8				+			72	72	V	P	GDAP c.818G>A (p.Arg273Gln)	CMT 2K	NO	Homozygous	YES (son)

9	+					+	22	7	V	P	POLG c.818G>A (p.Arg273Gln)	CPEO	L-DOPA [18], avoid valproic acid, use of ATQ3 [20]	Heterozygous	NO

10	+						58	10	V	P	MFN2 c.187A>C (p.Asn63His)	CMT 2A2A	NO	Heterozygous	NO

11						+	71	21	V	V	DDC c.73G>A (p.Glu25Lys)	AADC deficiency	Dopaminergic agonists, MAOIs, vitamin B6 [21]	Heterozygous	NO

12			+		+		53	10	V	V	CHMP2B c.581C>T (p.Ser194Leu).	ALS type 17	NO	Heterozygous	NO

13			+				49	4	V	V	C9orf72 c.80G>A (p.Arg27Gln)	ALS + FTD	NO	Heterozygous	NO

14	+						73	3	V	P	ELOVL5 c.327+1G>A	SCA 38	DHA [14]	Heterozygous	NO

15	+				+		74	–	V	V	PRPH c.421G>T (p.Asp141Tyr) + c.870+5A>G	ALS	NO	Compound heterozygous	YES (sister)

16	+						33	–	V	V	OPA1 c.1397C>T (p.Ala466Val)	DOA	NO	Heterozygous	NO

17	+					+	93	6	V	V	SETX c.3436A>G (p.Ser1146Gly)	.	NO	Heterozygous	NO

18						+	25	23	V	V	AUTS2 c.2972A>C (p.Asp991Ala)	.	NO	Heterozygous	NO

19	+						23	17	P/V	P for deafnessV for ataxia	MYO15A c.8003_8004insA(p.Thr2669Hisfs*43) + c.387A>C(p.Lys129Asn)	MYO15A	NO	Compound heterozygous	NO

20			+				63	3	P/V	V	VPS13A c.1115dup (p.Leu373Valfs*4) + VPS13C c.5209G>A (p.Ala1737Thr)	Juvenile PD (*parkin*)	NO	Compound heterozygous	NO

21			+				65	25	P	P	PRKN c.155del (p.Asn52Metfs*29) + c.823C>T (p.Arg275Trp)	Juvenile PD (*parkin)*	NO	Compound heterozygous	YES(father)

22				+			20	3	P	P	PRKN c.823C>T(p.Arg275Trp) + c.535-2A>C	Juvenile PD (*parkin*)	Trihexyphenidyl [16], Levodopa [22], DBS [23]	Compound heterozygous	YES(brother)

23			+				56	10	P	P	GRN c.1562G>A (p.Cys521Tyr)	FTD	NO	Heterozygous	NO

24			+				55	4	P	P	CYP27A1 c.1183C>T (p.Arg395Cys) + c.1214G>A (p.Arg405Gln)	CTX	CDCA [13]	Compound heterozygous	NO

25	+						34	20	P	P	SPG7 c.1A>G (p.Met1?) + c.1529C>T (p.Ala510Val)	SPG7	NO	Compound heterozygous	NO

26	+		+			+	29	6	P	P	ATP1A3 c.1877C>T (p.Thr626Met)	DYT12	Avoid triggers, use of flunarizin [12,24]	Heterozygous	Unknown(adopted)

27				+			31	5	P	P	CYP27A1 c.421G>T (p.Asp141Tyr)	CTX	CDCA [13]	Heterozygous	NO

28	+					+	32	32	P	P	PMM2 c.722G>C (p.Cys241Ser) + c.422G>A(p.Arg141His)	CDG-Ia	Acetazolamide [15]	Compound heterozygous	YES(sister case 29)

29	+					+	35	35	P	P	PMM2 c.722G>C (p.Cys241Ser) + c.422G>A(p.Arg141His)	CDG-Ia	Acetazolamide [15]	Compound heterozygous	YES(sister case 28)

30					+		57	–	P	P (for Rippling muscle disease)	CAV3 c.80G>A (p.Arg27Gln)	LGMD	NO	Heterozygous	YES(maternal cousin, aunt, uncles and grandfather)

31						+	32	27	P	P	THAP1 c.505C>T(p.Arg169*)	DYT6	GPi DBS [25]	Heterozygous	YES(mother, maternal grandmother and aunt)

32						+	29	29	P	P	KMT2B c.165del (p.Pro56Argfs*111)	DYT28	GPi DBS [19]	Heterozygous	NO


Crosses (+) indicate presence of a given phenotype in each patient; AADC (aromatic L-amino acid decarboxylase), ALS (Amyotrophic Lateral Sclerosis), ATQ3 (α-tocotrienol quinone), CDCA (chenodeoxycholic acid), CDG-Ia (Congenital Disorder of Glycosylation type Ia), CMT (Charcot-Marie-Tooth), CPEO (Chronic Progressive External Ophthalmoplegia), CTX (Cerebrotendinous Xanthomatosis), DHA (docosahexaenoic acid), DOA (Dominant Optic atrophy), DYT (dystonia), FTD (Frontotemporal Dementia), GPi DBS (Deep Brain Stimulation of the Globus Pallidus interna), LGMD (Limb-Girdle Muscular Dystrophy), MAOIs (monoamine oxidase inhibitors), MYO15A (autosomal recessive hearing loss, ataxia and polyneuropathy), P (Pathogenic), PD (Parkinson’s Disease), SPG (spastic paraplegia), V (Variant of Unknown Significance).

Establishing etiology enabled starting or modifying evidence-based treatment in 9 patients, in whom the variant identified was either considered pathogenic, or clinically interpreted as disease-causing. Treatments included conservative measures such as avoiding triggers in DYT12 cases [12]; prescribing supplements such as chenodeoxycholic acid in cerebrotendinous xanthomatosis [13], docosahexaenoic acid in SCA38 [14], acetazolamide in the congenital disorder of glycosylation Ia (CDG-Ia) due to mutations in the PMM2 gene [15]; as well as use of trihexyphenidyl for tremor treatment in juvenile Parkinson’s disease due to PRKN mutation [16]. In both patients presenting pathogenic variants in the PMM2 gene, and in one patient with POLG mutation, WES results allowed expansion of the phenotypic spectrum of the disorders, since dystonia had not been previously reported in the literature for these particular conditions [17,18]. For more information detailing treatments and outcomes, please see Supplementary Table 1.

**Supplementary Table 1:** **Treatments indicated based on current evidence and patient clinical outcomes.** SARA: Scale for the assessment and rating of ataxia.


PATIENT	MUTATION	DIAGNOSIS	TREATMENT	CLINICAL IMPROVEMENT

12	POLG c.818G>A (p.Arg273Gln)	Chronic Progressive External Ophthalmoplegia	Antioxidants, levodopa	Yes (mild, partial and transient)

21	DDC c.73G>A (p.Glu25Lys)	AADC (aromatic L-amino acid decarboxylase) deficiency	Rasagiline, ropinirole, vitamin B6	No

25	ELOVL5 c.327+1G>A	Spinocerebellar ataxia type 38 (SCA38)	Omega-3	Yes (reduction in SARA score, from 11 to 5 points)

4	PRKN c.823C>T(p.Arg275Trp) + c.535-2A>C	Juvenile Parkinson’s disease	Trihexyphenidyl	Yes (almost complete tremor resolution)

15	CYP27A1 c.1183C>T (p.Arg395Cys) + c.1214G>A (p.Arg405Gln)	Cerebrotendinous xanthomatosis	Chenodeoxycholic acid	No (insufficient doses due to lack of treatment availability)

18	CYP27A1 c.421G>T (p.Asp141Tyr)	Cerebrotendinous xanthomatosis	Chenodeoxycholic acid	Yes (mild, partial and transient)

17	ATP1A3 c.1877C>T (p.Thr626Met)	Dystonia 12	Avoidance of triggers	Yes (partial)

19	PMM2 c.722G>C (p.Cys241Ser) + c.422G>A(p.Arg141His)	Congenital Disorder of Glycosylation type Ia	Acetazolamide	Yes (mild and partial improvement in gait ataxia)

20	PMM2 c.722G>C (p.Cys241Ser) + c.422G>A(p.Arg141His)	Congenital Disorder of Glycosylation type Ia	Acetazolamide	No


In one patient who was adopted, family history was not available. Of the remaining 53, 12 had positive family history (22.6%). Of these, 9/32 (28.1%) presented positive test results combined with a positive family history, and 3/22 (13.6%) had a negative test result.We found no significant correlation between positive test result and age at disease onset or positive family history, probably due to the limited total number of patients. However, correlation was observed between positive test results and tremor phenotypes.

Major limitations to this analysis include the retrospective nature of the data collection, small sample size, lack of standardized diagnostic algorithm prior to WES, and heterogeneous criteria applied for indicating WES beyond treating physician preference, potentially generating significant selection bias as to which patients were tested and which were not. Furthermore, few patients were able to afford the study, therefore not all WES studies prescribed were performed. Because this was a consecutive cohort of patients studied with WES, and not a consecutive cohort of patients with atypical or combined phenotypes, diagnostic yield of WES could not be reliably assessed.

The ideal scenario in which to indicate WES is still hard to define. As a diagnostic tool it is still in early stages of development, with evolving discoveries, and only recently becoming more readily accessible. Further cohort studies may help determine which specific clinical characteristics will yield better test results. Currently, we believe WES should be considered early on, in patients with movement disorders presenting atypical or combined phenotypes, or early age at onset and positive family history, or in those lacking a clear diagnosis following thorough clinical evaluation by a trained specialist. The significant gap (***Table 1***) between symptom onset and definitive diagnosis based on WES, suggests earlier use could shorten time to start of treatment, as well as dampen patient diagnostic uncertainty.

Conversely, misinterpretations or VUS may decrease diagnostic accuracy. and lead to unnecessary testing or therapeutic trials, all of which can be considerably reduced when patients are evaluated by trained specialists.

## References

[1] Bonifati V. Will New Genetic Techniques Like Exome Sequencing and Others Obviate the Need for Clinical Expertise? Yes. Movement disorders clinical practice. 2017 Jan; 4(1): 36. DOI: 10.1002/mdc3.12438 30713946 PMC6353456

[2] Sethi KD, Lang AE. Will new genetic techniques like exome sequencing obviate the need for clinical expertise? No. Movement disorders clinical practice. 2017 Jan; 4(1): 39. DOI: 10.1002/mdc3.12443 30713947 PMC6353480

[3] Bertier G, Hétu M, Joly Y. Unsolved challenges of clinical whole-exome sequencing: a systematic literature review of end-users’ views. BMC medical genomics. 2016 Dec; 9(1): 1–2. DOI: 10.1186/s12920-016-0213-6 27514372 PMC4982236

[4] Klein CJ, Foroud TM. Neurology individualized medicine: when to use next-generation sequencing panels. InMayo Clinic Proceedings. 2017 Feb 1; 92(2): 292–305. Elsevier. DOI: 10.1016/j.mayocp.2016.09.008 28160876

[5] Trujillano D, Bertoli-Avella AM, Kandaswamy KK, Weiss ME, Köster J, Marais A, Paknia O, Schröder R, Garcia-Aznar JM, Werber M, Brandau O. Clinical exome sequencing: results from 2819 samples reflecting 1000 families. European Journal of Human Genetics. 2017 Feb; 25(2): 176–82. DOI: 10.1038/ejhg.2016.146 27848944 PMC5255946

[6] Krenn M, Wagner M, Strom TM, Auff E, Zimprich F. Diagnostic yield of whole-exome sequencing in neurological diseases. Journal of the Neurological Sciences. 2017 Oct 15; 381: 160. DOI: 10.1016/j.jns.2017.08.46828991672

[7] Stavropoulos DJ, Merico D, Jobling R, Bowdin S, Monfared N, Thiruvahindrapuram B, Nalpathamkalam T, Pellecchia G, Yuen RK, Szego MJ, Hayeems RZ. Whole-genome sequencing expands diagnostic utility and improves clinical management in paediatric medicine. NPJ genomic medicine. 2016 Jan 13; 1(1): 1–9. 10.1038/npjgenmed.2015.12 PMC5447450 28567303

[8] Valencia CA, Husami A, Holle J, Johnson JA, Qian Y, Mathur A, Wei C, Indugula SR, Zou F, Meng H, Wang L. Clinical impact and cost-effectiveness of whole exome sequencing as a diagnostic tool: a pediatric center’s experience. Frontiers in pediatrics. 2015 Aug 3; 3: 67. DOI: 10.3389/fped.2015.00067 26284228 PMC4522872

[9] Vissers LE, Van Nimwegen KJ, Schieving JH, Kamsteeg EJ, Kleefstra T, Yntema HG, Pfundt R, Van Der Wilt GJ, Krabbenborg L, Brunner HG, Van Der Burg S. A clinical utility study of exome sequencing versus conventional genetic testing in pediatric neurology. Genetics in Medicine. 2017 Sep; 19(9): 1055–63. DOI: 10.1038/gim.2017.1 28333917 PMC5589982

[10] Kuperberg M, Lev D, Blumkin L, Zerem A, Ginsberg M, Linder I, Carmi N, Kivity S, Lerman-Sagie T, Leshinsky-Silver E. Utility of whole exome sequencing for genetic diagnosis of previously undiagnosed pediatric neurology patients. Journal of child neurology. 2016 Dec; 31(14): 1534–9. DOI: 10.1177/0883073816664836 27572814

[11] Montaut S, Tranchant C, Drouot N, Rudolf G, Guissart C, Tarabeux J, Stemmelen T, Velt A, Fourrage C, Nitschké P, Gerard B. Assessment of a targeted gene panel for identification of genes associated with movement disorders. JAMA neurology. 2018 Oct 1; 75(10): 1234–45. DOI: 10.1001/jamaneurol.2018.1478 29913018 PMC6233854

[12] Marrodan M, Rossi M, Merello M. Rapid-onset dystonia-parkinsonism preceded by a single episode of subacute persisting hemiparesis: Expanding the ATP1A3-related disorders phenotype. Journal of the neurological sciences. 2018 Sep 15; 392: 44–5. DOI: 10.1016/j.jns.2018.07.002 30097153

[13] Salen G, Steiner RD. Epidemiology, diagnosis, and treatment of cerebrotendinous xanthomatosis (CTX). Journal of inherited metabolic disease. 2017 Nov; 40(6): 771–81. DOI: 10.1007/s10545-017-0093-8 28980151

[14] Manes M, Alberici A, Di Gregorio E, Boccone L, Premi E, Mitro N, Pasolini MP, Pani C, Paghera B, Orsi L, Costanzi C. Long-term efficacy of docosahexaenoic acid (DHA) for spinocerebellar ataxia 38 (SCA38) treatment: an open label extension study. Parkinsonism & related disorders. 2019 Jun 1; 63: 191–4. DOI: 10.1016/j.parkreldis.2019.02.040 30862453

[15] Martínez-Monseny AF, Bolasell M, Callejón-Póo L, Cuadras D, Freniche V, Itzep DC, Gassiot S, Arango P, Casas-Alba D, de la Morena E, Corral J. AZATAX: Acetazolamide safety and efficacy in cerebellar syndrome in PMM2 congenital disorder of glycosylation (PMM2-CDG). Annals of neurology. 2019 May; 85(5): 740–51. DOI: 10.1002/ana.25457 30873657

[16] Vernetti PM, Rossi M, Merello M. Parkin pleiotropy: extremely atypical phenotypes in patients with compound heterozygous mutations. Tremor and Other Hyperkinetic Movements. 2020; 10. DOI: 10.5334/tohm.55 32864185 PMC7427657

[17] Rossi M, Escobar AM, Ameghino L, Merello M. Expanding the phenotype of phosphomannomutase-2 gene congenital disorder of glycosylation: Cervical dystonia. Journal of the neurological sciences. 2017 Jul 15; 378: 52–4. DOI: 10.1016/j.jns.2017.04.037 28566178

[18] Rossi M, Escobar AM, Radrizzani M, Tenembaum S, Perandones C, Merello M. Dystonia in a Patient with Autosomal-Dominant Progressive External Ophthalmoplegia Type 1 Caused by Mutation in the POLG Gene. Movement disorders clinical practice. 2017 Mar; 4(2): 266. DOI: 10.1002/mdc3.12397 30838265 PMC6353407

[19] Zech M, Lam DD, Winkelmann J. Update on KMT2B-related dystonia. Current neurology and neuroscience reports. 2019 Nov; 19(11): 1–1. DOI: 10.1007/s11910-019-1007-y 31768667

[20] Stumpf JD, Saneto RP, Copeland WC. Clinical and molecular features of POLG-related mitochondrial disease. Cold Spring Harbor perspectives in biology. 2013 Apr 1; 5(4): a011395. DOI: 10.1101/cshperspect.a011395 23545419 PMC3683902

[21] Wassenberg T, Molero-Luis M, Jeltsch K, Hoffmann GF, Assmann B, Blau N, Garcia-Cazorla A, Artuch R, Pons R, Pearson TS, Leuzzi V. Consensus guideline for the diagnosis and treatment of aromatic l-amino acid decarboxylase (AADC) deficiency. Orphanet journal of rare diseases. 2017 Dec; 12(1): 1–21. DOI: 10.1186/s13023-016-0522-z 28100251 PMC5241937

[22] Kitada T, Asakawa S, Hattori N, et al. Mutations in the parkin gene cause autosomal recessive juvenile parkinsonism. Nature. 1998; 392(6676): 605–6089560156. DOI: 10.1038/33416 9560156

[23] de Oliveira LM, Barbosa ER, Aquino CC, Munhoz RP, Fasano A, Cury RG. Deep brain stimulation in patients with mutations in Parkinson’s disease–related genes: a systematic review. Movement disorders clinical practice. 2019 Jun; 6(5): 359–68. DOI: 10.1002/mdc3.12795 31286005 PMC6592794

[24] Masoud M, Prange L, Wuchich J, Hunanyan A, Mikati MA. Diagnosis and treatment of alternating hemiplegia of childhood. Current treatment options in neurology. 2017 Feb 1; 19(2): 8. DOI: 10.1007/s11940-017-0444-7 28337648

[25] Groen JL, Ritz K, Contarino MF, van de Warrenburg BP, Aramideh M, Foncke EM, van Hilten JJ, Schuurman PR, Speelman JD, Koelman JH, de Bie RM. DYT6 dystonia: mutation screening, phenotype, and response to deep brain stimulation. Movement disorders. 2010 Oct 30; 25(14): 2420–7. DOI: 10.1002/mds.23285 20687191

